# Case report: Novel mutations in the SPG11 gene in a case of autosomal recessive hereditary spastic paraplegia with a thin corpus callosum

**DOI:** 10.3389/fnint.2023.1117617

**Published:** 2023-03-24

**Authors:** Ji-Qing Duan, Hui Liu, Jia-Qiao Wu

**Affiliations:** ^1^Department of Intensive Care Unit, The Third People's Hospital of Chengdu, Affiliated Hospital of Southwest Jiaotong University, Chengdu, China; ^2^Department of Anesthesia, The Third People's Hospital of Chengdu, Affiliated Hospital of Southwest Jiaotong University, Chengdu, China

**Keywords:** hereditary spastic paraplegia, thin corpus, SPG11, gene mutation, case report

## Abstract

A 24-year-old man presented with insidious onset progressive gait disturbance and was finally diagnosed with autosomal recessive hereditary spastic paraplegia. Two novel mutations, including a frameshift mutation (c.5687_5691del) and a non-sense mutation (c.751C>T), were identified in the SPG11 gene of the patient through whole genome sequencing. The frameshift mutation of c.5687_5691del leads to a change in amino acid synthesis beginning with amino acid No. 1896 arginine and terminating at the 8th amino acid after the change (p. Arg1896MetfsTer8). The non-sense mutation (c.751C>T) causes the conversion of codon 251st encoding the amino acid Gln into a stop codon (p. Gln251Ter), resulting in premature termination of peptide synthesis. Although confirmation of compound-heterozygosity could not be performed, our findings enriched the phenotypic spectrum of SPG11 mutations related to hereditary spastic paraplegia.

## Background

A 24-year-old man presented with insidious onset progressive gait disturbance and was finally diagnosed with autosomal recessive hereditary spastic paraplegia. Two novel mutations, including a frameshift mutation (c.5687_5691del) and a non-sense mutation (c.751C>T), were identified in the SPG11 gene of the patient through whole-genome sequencing. The frameshift mutation of c.5687_5691del leads to a change in amino acid synthesis beginning with amino acid No. 1896 arginine and terminating at the eighth amino acid after the change (p. Arg1896MetfsTer8). The non-sense mutation (c.751C>T) causes the conversion of codon 251, encoding the amino acid Gln into a stop codon (p. Gln251Ter), resulting in premature termination of peptide synthesis. Although the confirmation of compound-heterozygosity could not be performed, our findings enriched the phenotypic spectrum of SPG11 mutations related to hereditary spastic paraplegia.

## Case presentation

**Figure 1:**
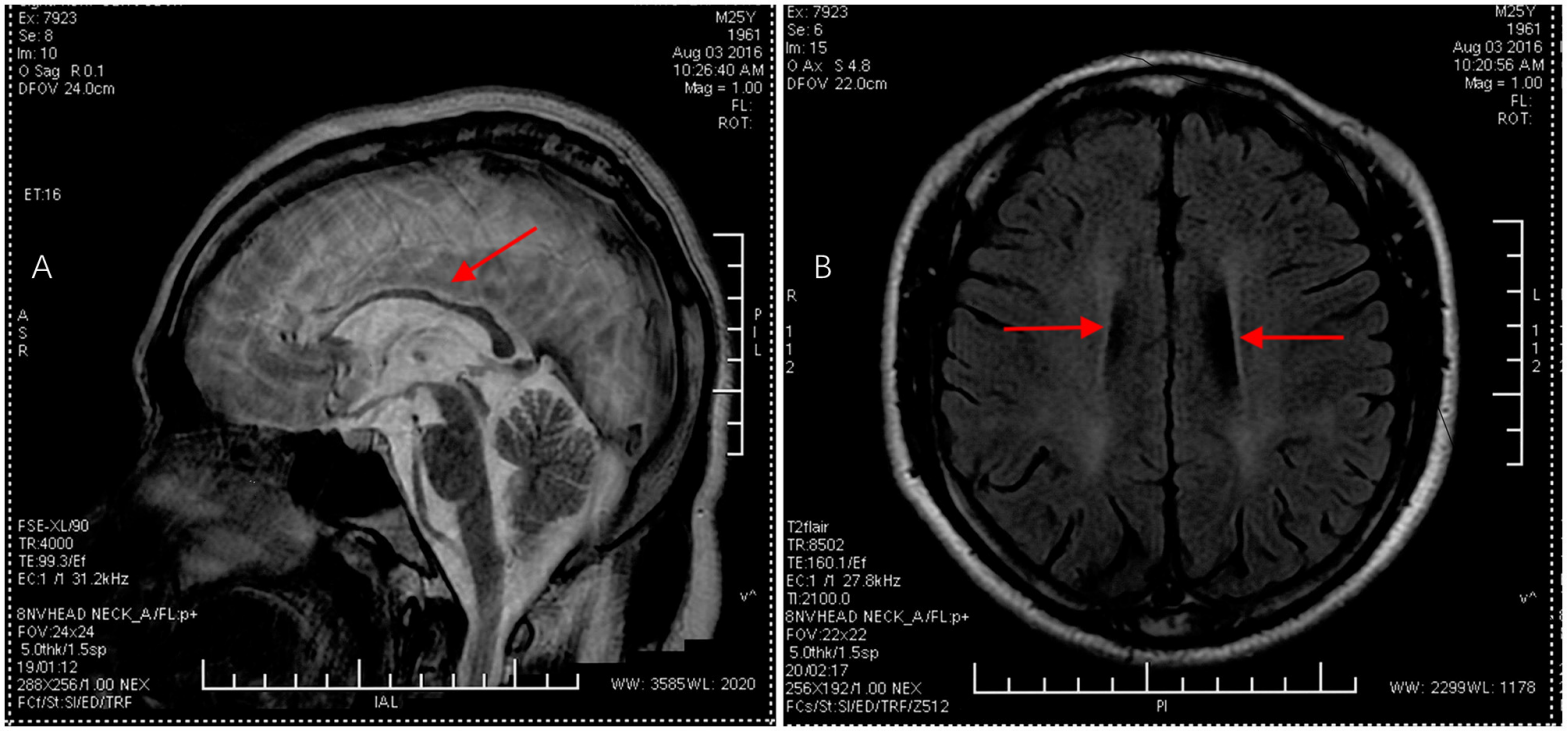
Brain MRI images in the proband. **(A)** Sagittal T2-weighted image displayed thinning of the corpus callosum, as indicated by the red arrow. **(B)** Axial T1-weighted image showed symmetrical periventricular white matter lesions, as indicated by the red arrow.

**Figure 2:**
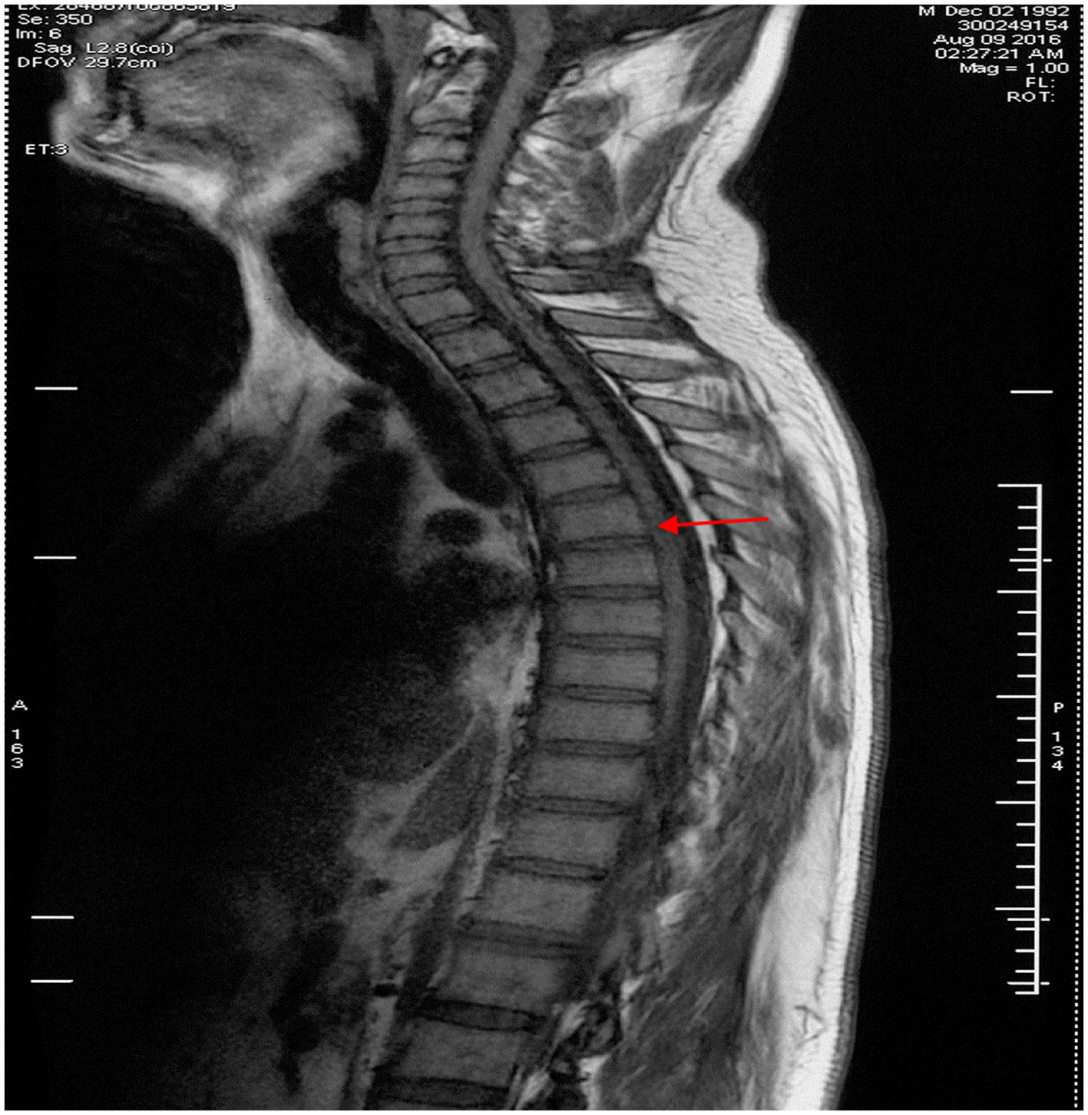
Thoracic spinal MRI image in the proband. Thoracic spinal MRI revealed thinning of the thoracic spinal cord with volume loss but no abnormal signal in the cord on sagittal T1-weighted imaging, as indicated by the red arrow.

**Figure 3:**
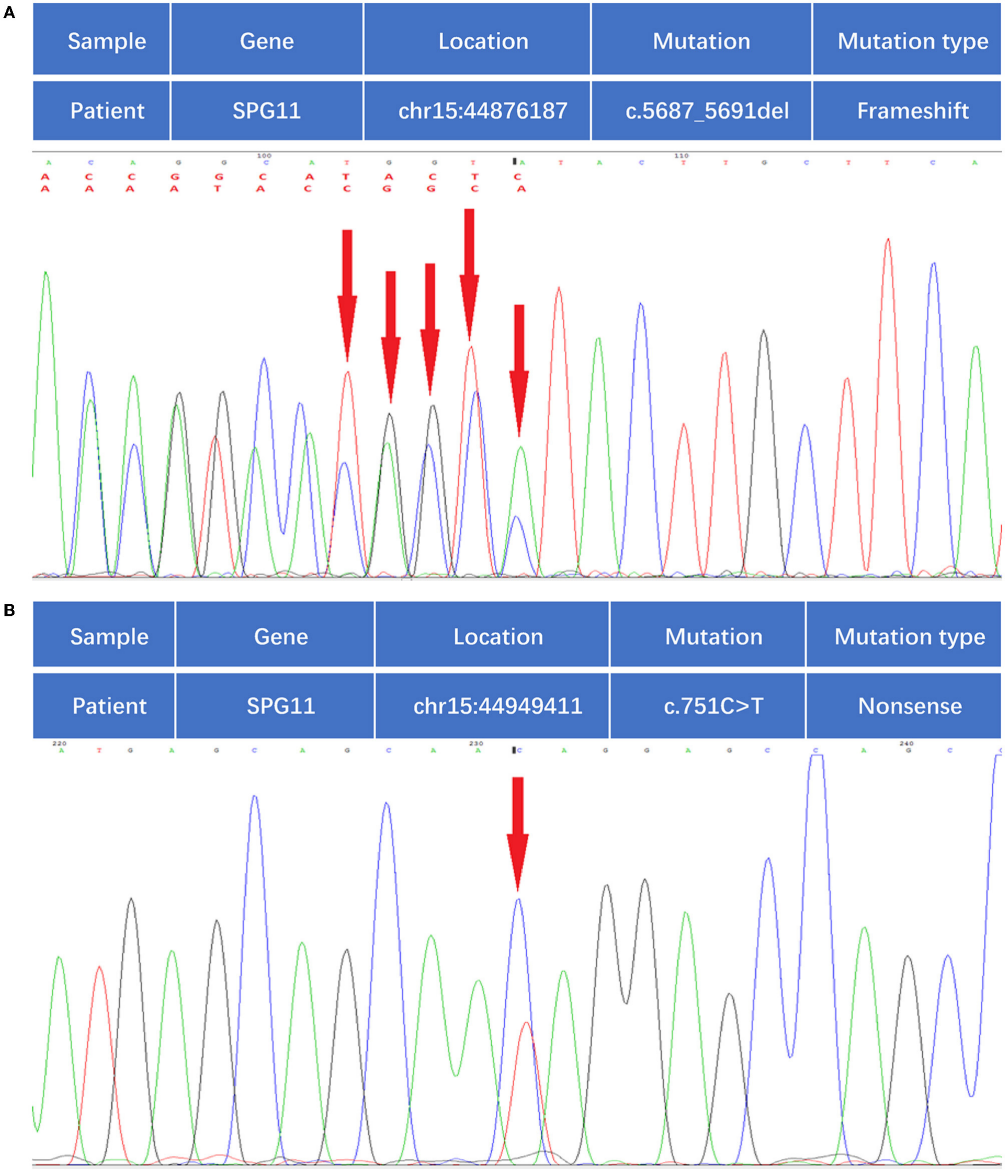
Whole-genome sequencing identified compound heterozygous mutations in the SPG11 gene. **(A)** Mutation of c.5687-5691del, as indicated by the red arrow. **(B)** Mutation of c.751C>T, as indicated by the red arrow.

A 24-year-old man born in a non-consanguineous family developed insidious onset progressive gait disturbance accompanied by weakness in the lower limbs for the past 4 years. The weakness of his lower extremities began distally and gradually progressed proximally. His performance at school was below average. Upon examination, his speech showed mild dysarthria, and his lower extremity strength was Medical Research Council (MRC) grade 4/5 with brisk reflexes and a bilateral extensor plantar response. He had pes cavus, normal muscle volume, bilateral foot drop, positive Babinski's sign, and scissor gait. There were no signs suggestive of upper limb problems. The scores of the mini-mental state examination (MMSE, normal range ≥27 points) and Montreal Cognitive Assessment (MoCA, normal range ≥26 points) were 29 points and 22 points, respectively, indicating mild cognitive impairment in the patient. Blood and cerebrospinal fluid (CSF) testing, including routine blood tests, serum immunologic tests, liver, and kidney function tests, as well as CSF biochemistry, immunologic tests, and oligoclonal bands, were all within the normal range. Magnetic resonance imaging (MRI) of the brain showed an extremely thin corpus callosum on sagittal views and symmetrical periventricular white matter lesions (Figures 1A, B). Thoracic MRI showed that the thoracic spinal cord was significantly thinner and reduced in volume (Figure 2). However, there was no obvious abnormal signal in the thoracic spinal cord on MRI images. No obvious abnormality was found in an electromyogram (EMG) or an electroencephalogram (EEG) of the patient. Somatosensory evoked potentials (SEPs) showed a significant decrease in the amplitude and central conduction velocity of the lower extremities. Whole-genome sequencing (WGS) was performed on the patient. A frameshift mutation (c.5687_5691del) and a non-sense mutation (c.751C>T) were identified in the SPG11 gene of the patient (Figure 3). The diagnosis of autosomal recessive HSP with a thin corpus callosum was finally confirmed according to the clinical manifestations and genetic test results of the patient (Patel et al., 2016; Zhang et al., 2016; Meyyazhagan et al., 2022). The Spastic Paraplegia Rating Scale (SPRS) was 16 points according to a previous study (Schüle et al., 2006). The patient received a daily dose of 60 mg edaravone and 0.5 g citicoline for ~2 weeks, and he was seen for follow-up visits once every 3 months. However, the weakness of his lower extremities and his gait disturbance was not effectively relieved.

## Discussion

HSP is a genetically heterogeneous group of neurodegenerative disorders characterized by progressive spasticity and lower extremity weakness as a result of retrograde axonal degeneration of the corticospinal tracts and posterior columns (Samaranch et al., 2008; Stevanin et al., 2008). HSP with a thin corpus callosum (HSP-TCC) is a distinguished subgroup of complicated forms and is often inherited as a recessive trait (Sperfeld et al., 2005; Hourani et al., 2009; Riverol et al., 2009). HSP-TCC has a high rate of disability, and most of the affected patients will become wheelchair-bound one or two decades after the onset of the disease (Stevanin et al., 2007). The clinical features of HSP begin primarily before the second decade of life. The estimated prevalence for HSP of all types ranges from 1:100,000 to 10:100,000, affecting diverse ethnic groups. The diagnosis of HSP is mainly based on clinical characteristics, neuroimaging, and gene testing (Shribman et al., 2019). The pathogenic mechanism, associated clinical characteristics, and neuroimaging abnormalities vary greatly, owing to different disease-causing genes, making it challenging to distinguish HSP from other genetic disorders associated with spasticity. Gene testing is now widely available, but there are limitations, and molecular diagnosis cannot be performed in most suspected cases. With a further understanding of the pathophysiological basis of individual HSP subtypes, opportunities are emerging to provide targeted molecular therapies and personalized medications (Shribman et al., 2019).

To date, 15 distinct loci associated with HSP-TCC have been identified: SPG 1, SPG 11, SPG 15, SPG 18, SPG 21, SPG 44, SPG 45 (65), SPG 46, SPG 47, SPG 49, SPG 54, SPG 56, SPG 63, SPG 67, and SPG 71 (Boukhris et al., 2010; Finsterer et al., 2012). In the current study, we identified two novel SPG 11 gene mutations: a frameshift mutation (c.5687_5691del) and a non-sense mutation (c.751C>T). SPG11-associated HSP is supposed to be the most frequent type of complicated autosomal recessive HSP, especially for patients with a thin corpus callosum and intelligence disorder (Paisanruiz et al., 2008; Meyyazhagan et al., 2022). The SPG 11 gene, which is located at chromosome 15q13-15, encodes the spatacsin protein that is primarily expressed in the pineal gland, cerebral cortex, cerebellum, and hippocampus (Paisanruiz et al., 2008; Samaranch et al., 2008; Panza et al., 2022). The genetic analysis of SPG11 revealed frequent truncating mutations altering the protein structure and function in the KIAA1840 gene (Lossos et al., 2006; Fink, 2013).

Among the mutations that have been reported in HSP, non-sense and frameshift mutations are the most frequent types described in SPG 11 (Liao et al., 2008; Cao et al., 2013; Tian et al., 2016; Du et al., 2018). To our knowledge, the mutation of SPG11 as seen in our case has never been reported in the literature. In our case, a frameshift mutation of c.5687_5691del (deletion of nucleotide number 5687_5691 in the coding region) was found in the SPG11 gene of the subject. The mutation resulted in a change in amino acid synthesis beginning with amino acid No. 1896 arginine and terminating at the eighth amino acid after the change (p. Arg1896MetfsTer8). A non-sense mutation of c.751C>T (nucleotide 751 in the coding region changes from C to T) was also noted, which causes the conversion of codon 251, encoding the amino acid Gln into a stop codon (p. Gln251Ter), resulting in premature termination of peptide synthesis. Such a mutation would bring about a truncated protein, resulting in a loss of the physiological function of the spatacsin protein (Stevanin et al., 2008). Both mutations can be pathogenic according to a joint consensus recommendation (Richards et al., 2015).

HSP induced by mutations of SPG 11 is characterized by early-onset progressive spasticity and weakness, a thin corpus callosum, and cognitive dysfunction. Approximately 79% of patients with HSP-TCC initially suffered from walking difficulties, and ~16% of patients first presented with symptoms of intellectual disability (Stevanin et al., 2008; Panza et al., 2022). In our case, the patient who presented with insidious onset progressive gait disturbance also had weakness in the lower limbs. A mild impairment of the cognitive function of the patient was also observed. Other symptoms of the patient included scissor gait, bilateral foot drop, and mild dysarthria. All the symptoms were consistent with previous studies (Liao et al., 2008; Cao et al., 2013; Tian et al., 2016; Du et al., 2018). Regrettably, the patient's father died in a car accident, his mother refused to undergo in-hospital genetic tests, and we failed to obtain his family pedigree. Although family segregation studies were not possible as explained, the phenotype and the characteristics of the variants detected indeed support their pathogenicity.

## Conclusion

We reported a rare case of HSP-TCC accompanied by two novel mutations of SPG11, and our findings enriched the phenotypic spectrum of SPG11 mutations. For patients presenting with HSP-TCC, we recommend direct testing for the SPG11 gene to establish the role of SPG11 mutation in these rare disorders.

## Data availability statement

The original contributions presented in the study are included in the article/supplementary material, further inquiries can be directed to the corresponding author.

## Ethics statement

Written informed consent was obtained from the individual(s) for the publication of any potentially identifiable images or data included in this article.

## Author contributions

J-QD wrote the initial draft (including substantive translation). HL revised the manuscript. J-QW approved the final version. All authors contributed to the article and approved the submitted version.

## References

[B1] Boukhris A. Feki I. Elleuch N. Miladi M. I. Boland-Augé A. Truchetto J.. (2010). A new locus (SPG46) maps to 9p21.2-q21.12 in a Tunisian family with a complicated autosomal recessive hereditary spastic paraplegia with mental impairment and thin corpus callosum. Neurogenetics 11, 441–448. 10.1007/s10048-010-0249-2 20593214

[B2] Cao L. Rong T. Y. Huang X. J. Fang R. Wu Z. Y. Tang H. D.. (2013). Novel SPG11 mutations in Chinese families with hereditary spastic paraplegia with thin corpus callosum. Parkinsonism Relat. Disord. 19, 367–370. 10.1016/j.parkreldis.2012.10.007 23121729

[B3] Du J. Hu Y. C. Tang B. S. Jiang H. Shen L. (2018). Identification of novel SPG11 mutations in a cohort of Chinese families with hereditary spastic paraplegia. Int. J. Neurosci. 128, 146–150. 10.1080/00207454.2017.1378878 28933964

[B4] Fink J. K. (2013). Hereditary spastic paraplegia: clinico-pathologic features and emerging molecular mechanisms. Acta Neuropathol. 126, 307–328. 10.1007/s00401-013-1115-8 23897027 PMC4045499

[B5] Finsterer J. Löscher W. Quasthoff S. Wanschitz J. Auer-Grumbach M. Stevanin G. (2012). Hereditary spastic paraplegias with autosomal dominant, recessive, X-linked, or maternal trait of inheritance. J. Neurol. Sci. 318, 1–18. 10.1016/j.jns.2012.03.025 22554690

[B6] Hourani R. El-Hajj T. Barada W. H. Hourani M. Yamout B. I. (2009). MR imaging findings in autosomal recessive hereditary spastic paraplegia. AJNR Am. J. Neuroradiol. 30, 936–940. 10.3174/ajnr.A1483 19193756 PMC7051668

[B7] Liao S. S. Shen L. Du J. Zhao G. H. Wang X. Y. Yang Y.. (2008). Novel mutations of the SPG11 gene in hereditary spastic paraplegia with thin corpus callosum. J. Neurol. Sci. 275, 92–99. 10.1016/j.jns.2008.07.038 18835492

[B8] Lossos A. Stevanin G. Meiner V. Argov Z. Bouslam N. Newman J. P.. (2006). Hereditary spastic paraplegia with thin corpus callosum: reduction of the SPG11 interval and evidence for further genetic heterogeneity. Arch. Neurol. 63, 756–760. 10.1001/archneur.63.5.756 16682547

[B9] Meyyazhagan A. Kuchi Bhotla H. Pappuswamy M. Orlacchio A. (2022). The puzzle of hereditary spastic paraplegia: from epidemiology to treatment. Int. J. Mol. Sci. 23, 7665. 10.3390/ijms23147665 35887006 PMC9321931

[B10] Paisanruiz C. Dogu O. Yilmaz A. Houlden H. Singleton A. (2008). SPG11 mutations are common in familial cases of complicated hereditary spastic paraplegia (HSP). Neurology 70, 1384–1389. 10.1212/01.wnl.0000294327.66106.3d 18337587 PMC2730021

[B11] Panza E. Meyyazhagan A. Orlacchio A. (2022). Hereditary spastic paraplegia: genetic heterogeneity and common pathways. Exp. Neurol. 357, 114203. 10.1016/j.expneurol.2022.114203 35970204

[B12] Patel S. Sethi P. K. Anand I. Batra A. Gupta P. (2016). Hereditary spastic paraplegia with a thin corpus callosum due to SPG11 mutation. Neurol. India 64, 171. 10.4103/0028-3886.173660 26755014

[B13] Richards S. Aziz N. Bale S. Bick D. Das S. Gastier-Foster J.. (2015). Standards and guidelines for the interpretation of sequence variants: a joint consensus recommendation of the American College of Medical Genetics and Genomics and the Association for Molecular Pathology. Genet. Med. 17, 405–424. 10.1038/gim.2015.30 25741868 PMC4544753

[B14] Riverol M. Samaranch L. Pascual B. Pastor P. Irigoyen J. Pastor M. A.. (2009). Forceps minor region signal abnormality “ears of the lynx”: an early MRI finding in spastic paraparesis with thin corpus callosum and mutations in the spatacsin gene (SPG11) on chromosome 15. J. Neuroimaging 19, 52–60. 10.1111/j.1552-6569.2008.00327.x 19040626

[B15] Samaranch L. Riverol M. Masdeu J. C. Lorenzo E. Vidal-Taboada J. M. Irigoyen J.. (2008). SPG11 compound mutations in spastic paraparesis with thin corpus callosum. Neurology 71, 332–336. 10.1212/01.wnl.0000319646.23052.d1 18663179

[B16] Schüle R. Holland-Letz T. Klimpe S. Kassubek J. Klopstock T. Mall V.. (2006). The Spastic Paraplegia Rating Scale (SPRS): a reliable and valid measure of disease severity. Neurology 67, 430–434. 10.1212/01.wnl.0000228242.53336.90 16894103

[B17] Shribman S. Reid E. Crosby A. H. Houlden H. Warner T. T. (2019). Hereditary spastic paraplegia: from diagnosis to emerging therapeutic approaches. Lancet Neurol. 18, 1136–1146. 10.1016/S1474-4422(19)30235-2 31377012

[B18] Sperfeld A. D. Baumgartner A. Kassubek J. (2005). Magnetic resonance investigation of the upper spinal cord in pure and complicated hereditary spastic paraparesis. Eur. Neurol. 54, 181–185. 10.1159/000090294 16352904

[B19] Stevanin G. Azzedine H. Denora P. Boukhris A. Tazir M. Lossos A.. (2008). Mutations in SPG11 are frequent in autosomal recessive spastic paraplegia with thin corpus callosum, cognitive decline and lower motor neuron degeneration. Brain 131, 772–784. 10.1093/brain/awm293 18079167

[B20] Stevanin G. Santorelli F. M. Azzedine H. Coutinho P. Chomilier J. Denora P. S.. (2007). Mutations in SPG11, encoding spatacsin, are a major cause of spastic paraplegia with thin corpus callosum. Nat. Genet. 39, 366. 10.1038/ng1980 17322883

[B21] Tian X. Wang M. Zhang K. Zhang X. (2016). Novel SPG 11 mutations in hereditary spastic paraplegia with thin corpus callosum in a chinese family. Can. J. Neurol. Sci. 43, 833–840. 10.1017/cjn.2016.17 27018819

[B22] Zhang L. Mcfarland K. N. Jiao J. Jiao Y. (2016). A case report of SPG11 mutations in a Chinese ARHSP-TCC family. BMC Neurol. 16, 87. 10.1186/s12883-016-0604-5 27256065 PMC4891852

